# Unlocking the Potential: Caloric Restriction, Caloric Restriction Mimetics, and Their Impact on Cancer Prevention and Treatment

**DOI:** 10.3390/metabo14080418

**Published:** 2024-07-30

**Authors:** Ulises Edgardo De-Leon-Covarrubias, Jose Juan Perez-Trujillo, Sheila Adela Villa-Cedillo, Alejandra Guadalupe Martinez-Perez, Carlos Roberto Montes-de-Oca-Saucedo, Maria de Jesus Loera-Arias, Aracely Garcia-Garcia, Odila Saucedo-Cardenas, Roberto Montes-de-Oca-Luna

**Affiliations:** Histology Department, Faculty of Medicine, Universidad Autonoma de Nuevo Leon (UANL), Monterrey 64460, Mexico; ulises.deleoncvr@uanl.edu.mx (U.E.D.-L.-C.); jperez.me0052@uanl.edu.mx (J.J.P.-T.); svilla.me0121@uanl.edu.mx (S.A.V.-C.); alejandra.martinezpr@uanl.edu.mx (A.G.M.-P.); carlos.montess@uanl.edu.mx (C.R.M.-d.-O.-S.); mdjesus.loeraars@uanl.edu.mx (M.d.J.L.-A.); aracely.garciagr@uanl.edu.mx (A.G.-G.); odila.saucedocr@uanl.edu.mx (O.S.-C.)

**Keywords:** cancer, caloric restriction, caloric restriction mimetics, metformin, aspirin, clinical trials

## Abstract

Caloric restriction (CR) and its related alternatives have been shown to be the only interventions capable of extending lifespan and decreasing the risk of cancer, along with a reduction in burden in pre-clinical trials. Nevertheless, the results from clinical trials have not been as conclusive as the pre-clinical results. Recognizing the challenges associated with long-term fasting, the application of caloric restriction mimetics (CRMs), pharmacological agents that mimic the molecular effects of CR, to harness the potential benefits while overcoming the practical limitations of fasting has resulted in an interesting alternative. This review synthesizes the findings of diverse clinical trials evaluating the safety and efficacy of CR and CRMs. In dietary interventions, a fast-mimicking diet was the most tolerated to reduce tumoral growth markers and chemotherapy side effects. CRMs were well tolerated, and metformin and aspirin showed the most promising effect in reducing cancer risk in a selected group of patients. The application of CR and/or CRMs shows promising effects in anti-cancer therapy; however, there is a need for more evidence to safely include these interventions in standard-of-care therapies.

## 1. Introduction

Cancer is a disease caused by the uncontrollable proliferation of cells that have lost the ability to respond to normal signals and gained the ability to invade tissues and organs, affecting their functions.

In 2020, the World Health Organization (WHO) reported 19 million new cases and estimated almost 10 million deaths around the world. Even with the most recent advances in surgery, chemotherapy (CT), and radiotherapy (RT), cancer remains the second most common cause of death worldwide, indicating the need for new complementary treatments against cancer.

Metabolic shifts are one of the representative hallmarks of cancer [1]. Elevated glucose consumption is one of the most representative changes in cancer cells. It allows for the high levels of ATP needed for the metabolic processes in neoplastic cells and serves as an intermediate metabolite for the synthesis of the nucleic acids and phospholipids required for cell division and the maintenance of tumor growth [2]. Furthermore, hexose can be derived from the pentose–phosphate pathway to generate NADPH and maintain the redox state in neoplastic cells [3].

Almost a hundred years ago, Otto Warburg hypothesized a mitochondrial dysfunction in cancer cells, which makes them dependent on high glucose consumption and aerobic glycolysis, a hypothesis named the Warburg effect [4]. Further studies have demonstrated correct mitochondrial functioning, which differs from this hypothesis.

Bearing this in mind, the modulation of glucose consumption in cancer cells can be highlighted as a complementary treatment. This approach might have the potential to decrease cell division, increase oxidative stress, and ultimately lead to cell death, making it a plausible co-therapy against cancer. Many pre-clinical studies have shown the feasibility of this therapy, which can reduce tumor growth and increase the sensitivity to CT and RT [5,6], but how can the modulation of glucose consumption be applied in a clinical context?

## 2. Methodology for Database Analysis

A comprehensive literature search using the PubMed, Google Scholar, and ClinicalTrials.gov databases was performed to retrieve information related to caloric restriction, caloric restriction mimetics, and their effect on cancer. The selection of the dietary interventions and the mimetics was made according to the results of pre-clinical studies and their applicability in humans. The keywords used for this search included “caloric restriction”, “ketogenic diet”, “fast mimicking diet”, “caloric restriction mimetics”, “metformin”, “rapamycin”, “aspirin”, and “resveratrol” in relation to cancer. Only research studies detailing in vivo, in vitro, and clinical trials published between 2010 and 2023 were included in this review. The web application Biorender.com was used for the creation of the figures.

## 3. Caloric Restriction

### 3.1. Definition and Cancer-Associated Molecular Pathways 

Caloric restriction (CR) is defined as a reduction in energy intake (<500 kCal) without incurring malnutrition [7]. The benefits of this dietary intervention in adults have been reported for years, and CR is the only reported intervention capable of extending lifespan; it is cardio-, osteo-, sarco-, and neuroprotective, and has been reported to be able to lower the incidence and progression of cancer [8,9]. However, a reduction in food consumption to achieve caloric restriction can be difficult for a healthy adult person and almost impossible for an oncologic patient, so alternative dietary regimens are necessary.

To overcome this, alternative regimens have been tested in cancer patients; among them are the ketogenic diet (KD), intermittent fasting (IF), and short-term starvation (STS).

The KD limits the consumption of carbohydrates and promotes the intake of fats; this induces ketosis through the mobilization of stored fatty acids for energy needs [10]. IF is a dietary intervention that limits the timing of caloric consumption, regardless of the content or number of calories [11]. STS is another dietary intervention that requires fasting for several days [12].

**Figure 1:**
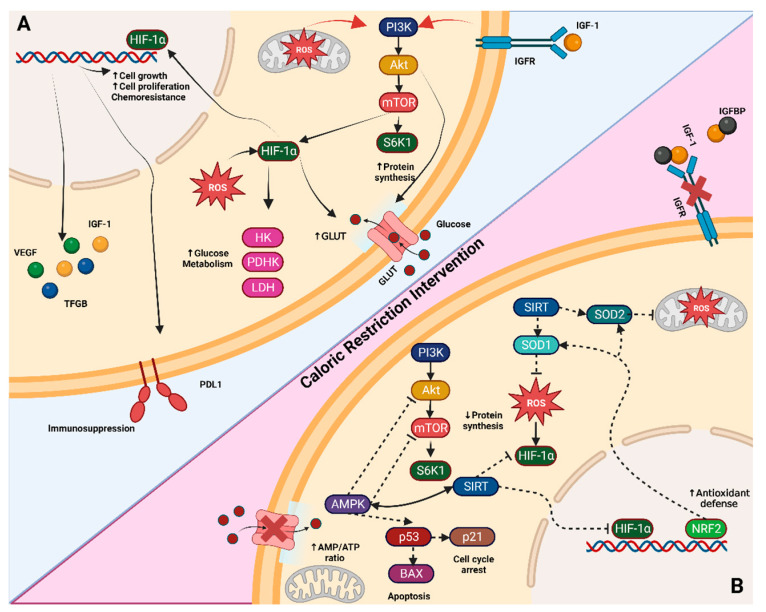
Tumor-altered pathways and CR molecular targets. (**A**) One of the commonly altered pathways in tumor cells is the PI3K/AKT/mTOR pathway, which leads to increased protein synthesis through S6K1 activation and the upregulation of HIF1α. HIF1α further promotes glycolytic intermediaries such as hexokinase (HK), pyruvate dehydrogenase kinase (PDK), and lactate dehydrogenase (LDH). Additionally, it induces the secretion of growth factors like insulin-like growth factor (IGF-1), transforming growth factor beta (TGF-β), and vascular endothelial growth factor (VEGF). These changes collectively enhance cell growth, proliferation, and chemoresistance. (**B**) When a caloric-restriction intervention is applied, such as glucose starvation, the increased AMP levels activate AMPK. This activation results in the inhibition of AKT and mTOR pathways and triggers a response mediated by p53 and p21, leading to interrupted protein synthesis and cell cycle arrest or apoptosis. The energy deficit also activates the SIRT pathways, which downregulates HIF1α, and the Nrf2 pathway, which enhances antioxidant defenses (e.g., superoxide dismutase SOD1 and SOD2) and reduces reactive oxygen species (ROS). Sky blue background shows tumor-altered pathways in non-caloric restriction (normal) diet, meanwhile red background shows the pathways during caloric restriction diet. Figure created with BioRender.com. Note: Arrows indicate activation, while dashed bar lines indicate inactivation.

No matter the dietary intervention, all of them have been shown to be tolerable to oncologic patients and have some benefits; this effect is related to the regulation of a variety of molecular pathways (Figure 1) [13,14,15].

#### 3.1.1. Energy Sensor Pathways: PI3K-AKT-mTOR and AMPK

The phosphoinositide 3-kinase (PI3K), AKT, mammalian target of rapamycin (mTOR) pathway (PIK3-AKT-mTOR) responds to the availability of nutrients, hormones, and growth factors, playing a crucial role in cell growth, metabolism, and proliferation [16]. AKT and mTOR can increase the transcription of glycolytic enzymes and glucose transporters (GLUTs), making them attractive targets for cancer therapy [17]. AKT and mTOR inhibitors are currently undergoing preclinical and clinical trials, underscoring the importance of regulating oncologic metabolism. As a nutrient-sensor mechanism, the PI3K-AKT-mTOR pathway can be regulated by both the depletion and increase in metabolites. CR modulates this pathway by activating AMP-activated protein kinase (AMPK), mediated by an increased AMP/ATP ratio in a glucose-limited environment [18]. AMPK activation leads to the phosphorylation of Rictor, resulting in the inactivation of the mTOR complex. Additionally, the phosphorylation of tuberous sclerosis complex 2 (TSC2) by AMPK further inhibits the mTOR complex, thereby increasing autophagy flux. This enhanced autophagy can be utilized to induce cell death in cancer cells [17,19].

#### 3.1.2. Sirtuins

Sirtuins (SIRTs) are a family of NAD-dependent deacetylase enzymes involved in various processes such as carbohydrate metabolism, stress response, inflammatory response, lifespan regulation, and tumor formation [20]. Consequently, they have become a focal point for research into diseases such as cancer, age-related conditions, rheumatic diseases, and metabolic syndrome. SIRTs function as transcriptional regulators by deacetylating histones and modulating the chromatin structure, thereby influencing the transcription of multiple genes, including FOXO, PGC-1α, and PPARα, among others [21]. In metabolic functions, SIRTs downregulate glycolytic enzymes through deacetylation and repress the key transcriptional inducer HIF-1α [22,23].

Furthermore, SIRTs contribute to antioxidant protection by increasing superoxide dismutase 2 (SOD2) activity, an enzyme in the mitochondrial matrix, which mitigates the expression of HIF-1α in response to cellular injury [24,25]. In the context of cancer, the shift from glycolytic to oxidative metabolism and the downregulation of glutaminolysis mediated by SIRTs act as an anti-Warburg effect, reducing tumor growth. Among the sirtuins, SIRT1, SIRT3, SIRT4, and SIRT6 have been identified as significant targets for anti-tumor treatments [26]; SIRT4 has been proposed as a tumor-suppressor protein, reducing glutamine entry into mitochondria in response to DNA damage [27,28].

However, SIRT1 is a controversial protein with dual roles; it may enhance tumor growth [29,30] or prevent tumorigenesis [31,32]. Therefore, further studies incorporating controlled variables of tumor growth variables are essential to elucidate its precise role.

The most frequently reported molecular targets of CR are AMPK and SIRTs, both being dependent on each other. The exact mechanism by which CR activates SIRTs remains unclear, but it is hypothesized that the increase in NAD+ by the metabolic shift from fermentation to respiration triggers the activation of the sirtuins enzymes [33,34]. This theory implies that cells unable to undergo this metabolic shift, such as some tumoral cells, do not benefit from this pathway.

#### 3.1.3. Nrf2

The nuclear factor erythroid 2-related factor 2 (Nrf2) is a transcription factor that regulates the expression of antioxidant enzymes in response to oxidative damage and inflammation [35]. Additionally, Nrf2 controls the production of reducing cofactor and multi-drug resistance-associated proteins, working as a protective mechanism against cell damage. The induction and overexpression of Nrf2 have demonstrated tumor-suppressive activity in chemically induced phase 2 tumorigenesis [36,37,38,39]. Nevertheless, in well-established tumors, overexpression and mutant variants of Nrf2 and downregulation of Keap1, the main inactivator of Nrf2, have been associated with poor prognosis in cancer [40]. This pro-tumorigenic effect is mediated by protection against reactive oxygen species (ROS) generated by RT and increased resistance to CT through the production of multi-drug resistance-associated proteins [41,42].

Not well described up to date, the carcinogenic protective effect of CR has been linked to the expression of Nfr2 [43], as evidenced by studies showing that Nfr2-knockout ($\mathrm{Nrf}2^{-/-})$ mice on a CR diet develop chemically induced tumors, whereas wild-type ($\mathrm{Nrf}2^{+/}$) mice on a CR diet do not [44]. Understanding how CR activates the NRF2 pathway and elucidating the dual role of Nfr2 in tumorigenesis requires further research for its safe application in clinical trials.

#### 3.1.4. GH/IGF-1 Axis

The growth hormone/insulin-like growth factor-1 (GH/IGF-1) is the main axis of growth in the body. Its primary function extends from development through adulthood until levels of this hormone decrease to a critical point known as “somatopause”, which leads to changes in body composition and cellular metabolism [44]. The main function of this axis is to increase the anabolic processes, such as protein synthesis and mitosis, while downregulating apoptosis. Growth hormone (GH) is secreted by the adenohypophysis under the regulation of hypothalamic growth hormone-releasing hormone (GHRH) and somatostatin. The liver is the main target, where it interacts with the growth hormone receptor (GHR), triggering IGF-1 release as the principal effector of this axis.

IGF-1 binds to its receptor (IGF1R), which is practically ubiquitous, exerting systemic body growth effects [45]. The binding of IGF-1 to IGF1R initiates the PI3K-AKT-mTOR pathway, among other important pathways such as the Ras/Raf/MEK/ERK/MAPK pathway, signaling nutrient availability of nutrients to initiate cell division.

Due to its mitogenic and anti-apoptotic effects, the GH/IGF-1 axis has garnered interest as a potential target for anti-cancer therapy. Although not considered an oncogene [46], elevated blood IGF-1 levels, and more importantly, local or peritumoral IGF-1 levels [47,48,49], have been associated with poor prognosis, increased tumor growth, and resistance to cytotoxic therapy. Consequently, antibodies and small molecules targeting IGF-1 and IGF1R have been developed and tested in pre-clinical and clinical models, showing the potential to delay tumor growth and increase sensibility to CT and RT [50,51]. CT in vivo models have demonstrated its ability to reduce IGF1 levels in rodent models, suggesting a plausible non-pharmacological intervention [52].

Nevertheless, clinical studies have not shown that a CR diet can reduce serum IGF-1 levels in patients, as observed in preclinical models [53,54]. Nonetheless, the effect of CR in reducing tumor burden persists, suggesting a possible non-IGF-1 mediated response or a reduction in tumor and peritumoral expression of IGF-1 or IGF1R.

## 4. Dietary Interventions and Their Anti-Tumorigenic Effect: Bench to Bedside

The anti-tumor/antiproliferative effects of caloric restriction have been widely proven in vitro, showing that multiple tumor cell lines are affected contrary to non-tumoral cell lines. The standard CR induction has been through glucose restriction (GR) in the media based on reports of dependence on the Warburg effect in different tumoral cell lines. Reducing glucose to 1 g/L and 0 g/L, compared to 4.5 g/L, has shown a decreased proliferative effect, mainly in tumor cell lines. Additionally, in prostate cancer (PC) [55] and triple-negative breast cancer (TNBC) [56] cell lines, GR has been shown to trigger an increase in ROS, leading to cell death.

Among the multiple effects of GR, disrupting the cell cycle to induce cell arrest [57,58], activating the AMPK pathway, downregulating telomerase activity [59,60], decreasing migration [58], inducing apoptosis [61], and necrosis-dependent death [62] are the principal mechanisms implicated in the antiproliferative effect. Consequently, GR has been proposed as an anti-tumoral therapy. Additionally, GR has been related to an increased sensitivity to CT [63,64], allowing for a dosage reduction in the clinical context.

In higher mammals, mouse models have demonstrated that various nutraceutical regimens can be translated to a human diet, showing similar effects to those reported in vitro. CR, fasting-mimicking diet (FMD), and low caloric intake (LCI) reduced tumor burden in a breast cancer (BC) mouse model, with CR showing the maximal effect [65]. Comparing CR against KD in a pancreatic ductal adenocarcinoma (PDCA) model, CR but not KD helped to reduce tumor growth and mortality [66]. This outcome could be explained by the increased availability of lipids in KD, which can be used as fuel for energy generation and anabolic reactions.

Other outcomes found in CR diets as a treatment against cancer include an increase in tumor-infiltrating lymphocyte (TIL) CD8+ and PD-1 expression [67], a reduction in myeloid-derived suppressor cells [68], and tumor-forming stem cells. Additionally, CR decreases poor prognosis markers such as IGF1, pAKT, and PI3K [69] and induces changes in the gut microbiome linked to anti-tumor effects [70]. Incorporating a nutraceutical regimen like CR or KD with CT has reduced tumor growth and relapse and improved the survival rate [67,71].

In a clinical context (Table 1), the applicability of a CR diet in oncologic patients is controversial due to the potential adverse effects of significant body weight loss. Therefore, most clinical trials have used a body mass index (BMI) between 18.5 and 27.5 to mitigate the risk of induced cachexia. Nevertheless, the use of CR diets has been reported as well or moderately tolerated in most cancers [13,14,15,72], except in head and neck squamous cell carcinoma (HNSCC), where not even enteral nutrition was tolerated (72). The location and compressive effects of the tumor mass can be considered the principal problem for tolerability [73].

Another topic to consider is the type of dietary intervention, with FMD being the first option, followed by KD and CR last. FMD has been considered the most cost-effective and applicable because it does not completely restrict food intake, the food is the most tolerable when compared to KD, and it has a shorter fasting period compared to CR. This makes it the most approachable nutraceutical intervention against cancer.

Even though most of these clinical trials have only reached phase 1 with a limited number of patients, the outcomes are promising as complementary therapy regimens. KD is not well tolerated, with low adherence, and there were no changes in the overall survival (OS) and partial response (PR) for the small number of patients. There was an increase in serum carbonylated proteins after a combination of CT/RT with KD [74], presumably due to increased oxidative stress and partial cell death.

The results from clinical trial outcomes have been further validated in preclinical models to minimize the concomitant variables, with findings consistently indicating that dietary restrictions render highly proliferative tumor cells more susceptible to oxidative damage [56,57]. This is due to the reduction in glucose, a precursor of NADPH, the principal co-factor for many antioxidant systems and enzymes, rendering the cells unable to control oxidative damage and more susceptible to DNA damage caused by CRT [75].

Among other beneficial effects of dietary interventions is the increased endurance of healthy cells to cytotoxic drugs and radiotherapy. For example, CR has been reported to induce cell cycle arrest in the G0/G1 phases, enabling cells to undergo DNA repair more efficiently and diminishing DNA damage by CRT [58]. In contrast, tumoral cells, which have an altered cell cycle, are unable to repair DNA, leading to cell death [76]. Additionally, this protective effect can diminish myeloid suppression and secondary tumor formation from CT/RT damage. A pilot study in patients with diffuse large B-cell lymphoma on short-term calorie reduction (SCR) showed an increase in leukocyte count after the first intervention compared to the comparison group, but no difference was observed after the second [72]. Two more extensive studies with different neoplasias, one not randomized and one randomized, evidenced that inducing CR at least one hour prior to or after a CT round reduced leukocyte DNA CT-associated damage compared to the non-fasting group [13,77].

As previously reviewed, dietary interventions not only act directly on tumor glucose metabolism but also alter other metabolic pathways not directly linked to carbohydrate metabolism. This can be seen in a report on PC patients, where CR implementation increased IGFBP3 serum levels and tended to lower insulin serum levels [15]. The insulin/IGF1 growth axis has been implicated as a poor prognostic marker, not as an oncogenic pathway, but as a proliferative additive to the unregulated tumor cell signal [47,52]. IGFBP3 is the main IGF-transporter protein in the blood, where it forms a complex that stabilizes and enhances the half-life of insulin-like growth factors. It also reduces their interaction with IGFR, thus blocking activation and reducing mitogenic effects. Decreased IGFBP3 levels have been implicated as a risk factor in multiple neoplasias, making it an interesting target in oncologic treatment [78].

Other metabolic changes associated with dietary interventions combined with conventional anti-tumoral therapies include reduced serum glucose levels and a decrease in insulin, IGF1, and leptin [15,77]. Many cancer patients receive corticosteroids as prophylactic treatment against CRT side effects, but steroids can increase glucose and insulin levels, triggering growth signals in residual tumor cells as a collateral effect. Clinical trials have shown that CR can reduce both glucose and insulin levels in patients, regardless of the use of steroids [79], highlighting the potential of nutraceutical interventions in regulating not only the tumoral but overall metabolism, potentially improving the patient’s health outcomes.

The regulation of caloric intake in pre-clinical models has shown the capability to modulate the tumor immune response, improving cytotoxic responses against the immunosuppressive tumor microenvironment [67,68]. In oncologic patients, stimulating the immune response is a primary goal of immunomodulators like anti-PDL1. Therefore, the immune-regulatory function of CR could be beneficial in enhancing the anti-tumor response with fewer side effects than immunomodulatory drugs.

A phase 2 non-randomized study that included multiple types of cancer patients on FMD demonstrated that this dietary intervention could reduce monocytic myeloid-derived suppressor cells CD14+HLA-DR- and CD14+PD-LD+, both reported as highly suppressive subsets, while upregulating CD8+PD-1+CD69+-activated T cells, cytolytic natural killer (NK) cells, and CD3+CD25+ T cells, all reported as anti-tumor effectors [80]. This immunoregulatory effect is not only attributed to the cell phenotype, as another trial showed that KD was able to decrease TNF-a and increase IL-10 [81]. IL-10, an anti-inflammatory cytokine, has been reported as a potent inducer and activator of CD8+ T cells, having anti-tumoral properties that can be targeted [82].

**Table 1:** Caloric restriction and its applicability in anti-cancer trials.

Study Design	Type(s) of Cancer	SOC	Intervention	Outcomes	REF.
Phase 2. Randomized, controlled, observer-blind	HER- BC	CT with dexamethasone	Fast-mimicking diet	There is no difference in pCR. The toxicity was partially reduced after FMD without the use of dexamethasone. Increase in the MP. Reduction in glucose, insulin, IGF1, and DNA leukocyte damage.	[77]
Phase 1, non-randomized	Advanced NSCLC and PaCa	CRT	Ketogenic diet	No tolerability of the intervention. Not enough patients to analyze overall survival nor partial response. Increase in carbonylated proteins in serum as an indicator of increased oxidative stress.	[74]
Phase 1, non-randomized	HNSCC	CRT	Ketogenic diet	Poor tolerability of the diet. Not enough patients to analyze overall survival nor partial response. No evident changes in carbonylated proteins nor GSH as indicators of increased redox stress.	[73]
Phase 1, non-randomized	Multiple	CT	Short-term fasting	The intervention was well tolerated by all participants.Reduction in blood leukocyte damage after 24 h of fasting.	[13]
Pilot study, randomized, controlled	PC	Sx or active surveillance	Caloric restriction	The restriction diet was well tolerated. Increase in serum IGFBP-3 levels, along with insulin and C-peptide.	[15]
Phase 1, single arm	Multiple	SOC	Fast-mimicking diet	FMD along with the SOC decreased glucose, insulin, and IGF1 levels in serum. There was also a reduction in myeloid-derived suppressor cells and PD+ cells. In tumor and blood, there was an increase in CD8+, NK, and macrophage infiltration.	[80]
Phase 1, randomized, not blind	BC	CT	Ketogenic diet	Dietetic intervention reduced serum TNF-a and insulin levels. Also, CT plus KD reduced tumor size and TNM compared to control.	[81]
Pilot study, randomized, blind	DLBCL	CT + biologic	Short-term caloric reduction	The intervention was safe and feasible. The intervention improved hematological parameters.	[72]
Pilot study, randomized, cross-over	BC and OC	CT	Short-term fasting	Short-term fasting is feasible and reduces chemotherapy side effects, improving quality of life.	[83]
Phase 1, randomized, controlled	BC	SOC	Ketogenic diet	Ketogenic diet improved quality of life, but not in biomarkers after 12 weeks of intervention.	[84]

BC: breast cancer, BTC: biliary tract cancer, CC: Cervix Cancer, CRC: colorectal cancer, CRT: chemoradiotherapy, CT: chemotherapy, DLBCL: Diffuse Large B Cell Lymphoma, EC: endometrial cancer, FMD: fast-mimicking diet, FTC: follicular thyroid cancer, GC: Gastric Cancer, GSH: Glutathione, IGF: insulin-like growth factor, IGFBP-3: IGF Binding Protein 3, MP: Miller–Payne, NSCLC: non-small cell lung cancer, OC: ovarian cancer, OS: overall survival, PaCa: Pancreatic Cancer, PC: prostate cancer, pCR: pathological complete response, PR: partial response, SOC: standard of care therapy, Sx: surgery, TNBC: triple-negative breast cancer.

## 5. Caloric Restriction Mimetics (CRM)

### 5.1. Definition

**Figure 2:**
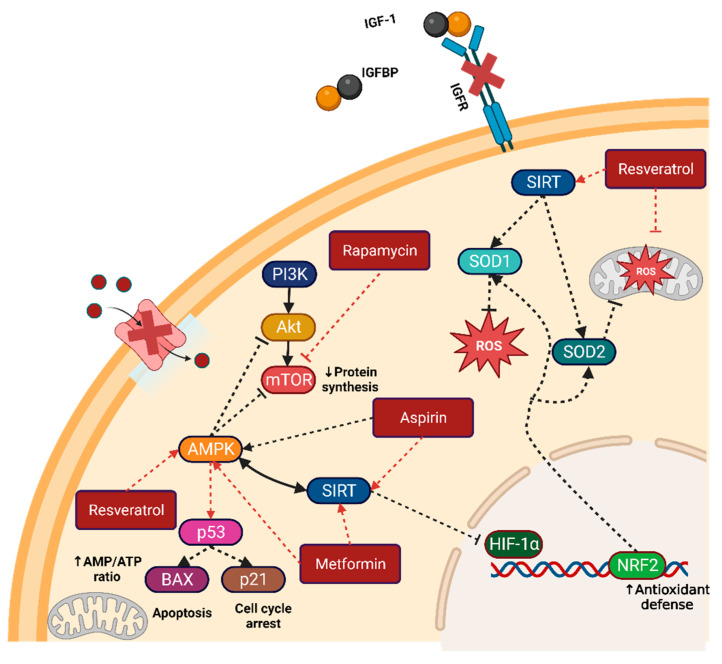
Caloric restriction mimetics and their targets. Metformin, aspirin, resveratrol, and rapamycin are the most common CRMs used in preclinical and clinical research. Their principal target is the direct or indirect activation of the master regulators AMPK and SIRT. Activation of these master regulators can lead to the inactivation of key growth and division pathways such as PI3K/AKT/mTOR and HIF1α. Figure created with BioRender.com. Note: arrows indicate activation, while dashed bar lines indicate inactivation.

Considering the difficulties oncologic patients face in maintaining a CR diet, pharmacological therapies have been developed to mimic the effects of CR interventions. Caloric restriction mimetics (CRM) are a variety of compounds that replicate the biochemical and signaling pathways triggered by CR, such as autophagy induction, AMPK activation, and SIRT expression, among other pathways. Examples of the most studied CRM and their anti-cancer effects include metformin, rapamycin, aspirin, and resveratrol and its by-products [8] (Figure 2).

#### 5.1.1. Metformin

Metformin is a biguanide molecule extensively used as an antidiabetic drug, primarily known as an insulin-sensitizer drug; it exerts multiple effects on most cells and their organelles [85]. The main effect on the mitochondria is that it inhibits complex I electron transfer, reducing NADH oxidation and thereby preventing ATP production [86]. The changes in the AMP/ATP ratio lead to activation of the AMPK pathway [59]. In tumor cells, AMPK activation can repress the PI3K/AKT/mTOR pathway, thereby inhibiting cell growth and tumorigenesis.

In preclinical studies, metformin has demonstrated the ability to induce cell cycle arrest and promote cell senescence [87]. Additionally, intrinsic apoptosis and autophagy-induced cell death have also been reported as anti-tumoral mechanisms of this drug [88]. Metformin also increases the activation of CD8+ lymphocytes and decreases T-reg levels [89], enhancing the anti-tumor immune response. Consequently, metformin has become a target of interest in oncology (Table 2).

A 2014 retrospective cohort study of 65,754 patients in Taiwan compared the effects of metformin therapy to other antidiabetic drugs used either alone or in combination. The analysis revealed that metformin reduced cancer risk in a dose-dependent manner, regardless of whether used alone or in combination, highlighting the growing interest in its anti-tumorigenic effects [90].

In non-diabetic women with operable stage I/II breast cancer, short-term neoadjuvant treatment with metformin, although not affecting tumor size, led to decreased Ki67 and phosphodiesterase 3 (PDE3B) staining in postoperative samples. It also resulted in reductions in weight, BMI, and glucose levels [91,92]. Metformin usage additionally increased transcriptomic signatures of TNFR1 pathway genes and TUNEL-positive cells [92].

In non-diabetic endometrial cancer patients, neoadjuvant use of metformin likewise decreased Ki67 staining along with insulin, IGF1, IGFBP7, and the surrogate marker of mTOR activation S6 ribosomal protein (Ps6) [93,94,95]. Also, they identified an increased activation of AMPK and p27, with subsequent reduction in cyclin D1 expression and cell cycle arrest [93].

For other cancer types, such as colorectal cancer (CRC), the addition of metformin to classical chemotherapy increased overall survival (OS) up to 7 months in obese patients without metabolic complications [94]. In non-small cell lung cancer (NSCLC) patients randomized to receive standard therapy with or without metformin, the combination of both therapies increased 1-year progression-free survival (PFS) to 45% compared to 15% in other trials without metformin, demonstrating the benefit of adding metformin to standard therapy [96].

Nevertheless, other studies have not shown benefits from adding metformin to the standard of care CT (SOC). These include studies on metastatic pancreatic [97] and breast cancer [98,99], advanced NSCLC [100,101], and castration-resistant prostate cancer [102]. This raises questions about whether metformin’s effects are primarily beneficial in the early stages of tumorigenesis, where glucose and growth signals are crucial for initial establishment and exponential growth.

**Table 2:** Metformin and its application in anti-cancer therapies.

Study Design	Type(s) of Cancer	SOC	CRM	Outcomes	REF.
Pilot study, randomized, non-blinded	BC	Sx	Metformin 1 g/12 h	Preoperative use of metformin showed no decrease in tumor size but a significant reduction of Ki-67 and PDE3B cells.	[91]
Population-based retrospective cohort study	None	None	Metformin	The usage of metformin monotherapy or in combination with other antidiabetic drugs reduced the incidence of cancer in type 2 diabetic patients.	[90]
Pilot study, single arm	EC	Sx	Metformin 2 g/d	Preoperative use of metformin decreased Ki-67 and Ps6 staining and reduced serum levels of glucose and IGF1. It decreased proliferative markers ERK1/2 and Cyclin D1.	[93]
Phase 2, randomized	NSCLC	CT	Metformin 2 g/d	Concomitant use of metformin increased progression-free survival by 32% at one year compared to control.	[96]
Phase 2, randomized	CC	CRT	Metformin	Metformin increased 2-year disease-free survival by up to 67% compared to 33% in the controls.	[103]
Phase 2, randomized, double-blind, placebo-controlled	BC	Sx	Metformin 850 mg/12 h	Overall, no difference in proliferation rates between groups. In subgroup analysis, metformin showed a heterogeneous effect dependent on insulin resistance.	[104]
Phase 2, randomized, placebo-controlled	BC	CR	Metformin 850 mg/12 h	There is no difference in progression-free survival nor overall response.	[99]
Phase 2, randomized	NSCLC	CR	Metformin 1000 mg/12 h	Overall, there was no difference in risk of progression or death compared to control. In high fluorodeoxyglucose-uptake cancers, the addition of metformin to CT decreased the risk of progression and death.	[105]

BC: breast cancer, BTC: biliary tract cancer, CC: Cervix Cancer, CRC: colorectal cancer, CRT: chemoradiotherapy, CT: chemotherapy, DLBCL: Diffuse Large B Cell Lymphoma, EC: endometrial cancer, FMD: fast-mimicking diet, FTC: follicular thyroid cancer, GC: Gastric Cancer, GSH: Glutathione, IGF: insulin-like growth factor, IGFBP-3: IGF Binding Protein 3, MP: Miller–Payne, NSCLC: non-small cell lung cancer, OC: ovarian cancer, OS: overall survival, PaCa: Pancreatic Cancer, PC: prostate cancer, pCR: pathological complete response, PR: partial response, SOC: standard of care therapy, Sx: surgery, TNBC: triple-negative breast cancer.

#### 5.1.2. Rapamycin and Its Analogs

Rapamycin, also known as Sirolimus, is the most studied CRM. It is a macrolide compound used as an immunosuppressant in solid organ transplants. The main function of rapamycin is the inhibition of the mTOR complex through its binding to the FK-binding protein 12 (FKBP12) and subsequently binding to mTOR Complex 1 (mTORC1), inhibiting its nutritional and proliferative effects [106]. Due to adverse effects such as edema, nausea, hypertriglyceridemia, anemia, and diabetes-like symptoms, the use of rapamycin is somewhat limited. Nevertheless, Temsirolimus and Everolimus, both rapamycin analogs (rapalogs), tend to have fewer adverse effects compared to rapamycin.

In clinical settings, Everolimus is FDA-approved as a complementary therapy for treating postmenopausal women with ER+HER- locally advanced or secondary BC (Table 3). The addition of rapalogs to aromatase inhibitors therapy, or their use as monotherapy, has resulted in varying increases in PFS, with reported durations of 22 months [107], 8.4 months [108], and 6.4 months [109]. The significant difference in PFS between studies must be carefully analyzed, as the discrepancy may be attributed to variations in the patient’s clinical history. The Everolimus dosage and experimental design could explain these variations and guide the effective use of rapalogs.

In follicular thyroid cancer, the addition of Everolimus led to better disease management and fewer adverse reactions compared to other treatments, such as Tyrosine Kinase Inhibitors (TKI). Notwithstanding, this was not observed in anaplastic thyroid cancer patients, highlighting the variability in cancer subtypes and their response to treatment [110]. In advanced biliary tract cancer (ABTC) patients, first-line therapy with Everolimus showed a median PFS of up to 5.5 months compared to the historical SOC platinum-gemcitabine of 8 months. This suggests the potential feasibility of adding mTOR inhibitors to increase PFS and OS [111].

In metastatic colorectal cancer, the addition of rapalogs to CT and Bevacizumab modestly increased PFS by 20% up to 6 months. Interestingly, the best response was observed in patients with mutations in PTEN and PIK3CA proteins [112]. Similarly, in HNSCC, a beneficial effect in a subgroup of PTEN mutation was observed after adding rapalogs [113], making plausible the selection of patients who might show the best response to mTOR inhibitors.

Nonetheless, clinical trials in patients with triple-negative breast cancer (TNBC) [114], HER2-BC [115,116], and prostate cancer [117,118] have not demonstrated additional benefits from incorporating Everolimus into standard therapy. This underscores how specific groups of patients with particular tumor characteristics may benefit from using rapalogs as a complementary regimen.

**Table 3:** Rapamycin and rapalogs and their application in anti-cancer therapies.

Study Design	Type(s) of Cancer	SOC	CRM	Outcomes	REF.
Phase 2, non-randomized	FTC	None	Everolimus 10 mg/d	Increased stable disease and overall survival compared to other interventions with fewer side effects.	[110]
Phase 2, single arm	BTC	None	Everolimus 10 mg/d	Median progression-free survival of 5.5 months and median OS of 9.5 months	[111]
Phase 2	CRC	CT+ biologic	Everolimus	The addition of Everolimus to standard therapy increased progression-free survival by 20% at 6 months. The subgroup of patients with PTEN mutations might show a better response.	[112]
Phase 2, single arm	BC	Aromatase inhibitor	Everolimus 10 mg/d	Everolimus and aromatase inhibitor (Exemestane) bitherapy increased progression-free survival by up to 6 months compared to aromatase inhibitor monotherapy.	[109]
Phase 2, randomized, double-blind, placebo-controlled	TNBC	CT	Everolimus 5 mg/d	Neoadjuvant addition of Everolimus to CT did not increase pathological complete response compared to placebo and added toxicity.	[114]
Phase 2, randomized	PC	Sx	Everolimus 5 mg/d vs. 10 mg/d	No difference in mTOR markers, nor PSA pre- and post-operative with the intervention.	[117]
Phase 2, randomized	HER2-BC	CT	Everolimus 5 mg/d	No difference in progression-free survival and overall survival compared to monotherapy alone.	[115]
Phase 2, non-randomized	FTC	None	Everolimus 10 mg/d	Increased stable disease and overall survival compared to other interventions with fewer side effects.	[110]

BC: breast cancer, BTC: biliary tract cancer, CC: Cervix Cancer, CRC: colorectal cancer, CRT: chemoradiotherapy, CT: chemotherapy, DLBCL: Diffuse Large B Cell Lymphoma, EC: endometrial cancer, FMD: fast-mimicking diet, FTC: follicular thyroid cancer, GC: Gastric Cancer, GSH: Glutathione, IGF: insulin-like growth factor, IGFBP-3: IGF Binding Protein 3, MP: Miller–Payne, NSCLC: non-small cell lung cancer, OC: ovarian cancer, OS: overall survival, PaCa: Pancreatic Cancer, PC: prostate cancer, pCR: pathological complete response, PR: partial response, SOC: standard of care therapy, Sx: surgery, TNBC: triple-negative breast cancer.

#### 5.1.3. Aspirin

Aspirin is a nonsteroidal anti-inflammatory drug (NSAID) whose main effect is the inhibition of cyclooxygenases (COX), suppressing the production of prostaglandins and thromboxanes, thereby dampening the inflammatory response. Besides its anti-inflammatory effects, aspirin and its salicylate derivatives are increasingly being considered as CRMs [119]. Further experiments have shown that aspirin inhibits the dephosphorylation of AMPK, maintaining high levels of this molecule and dampening the activation of anabolic pathways such as PI3K-AKT-mTOR [120].

The anti-carcinogenic effects of aspirin in the development of CRC have already been well-documented and are related to its COX inhibitor properties (Table 4). As such, aspirin has been associated with reduced CRC risk in Lynch syndrome patients [121] but not in non-colorectal cancers [122]. A retrospective cohort study also showed that aspirin reduces CRC risk in healthy patients when used before the age of 70 and for a duration of at least 5 years [123]. In a pilot study of healthy patients who consumed aspirin, no difference in the expression and catabolism of prostaglandins was observed, but there was a reduction in the expression of pS6 protein, the main target of the mTOR pathway, implicating that the protective effect of aspirin could be related to its CRM properties [124].

In non-CRC cancers, aspirin has shown various effects worth mentioning. In PC retrospective studies, aspirin use has been associated with a decrease in prostatic-specific antigen (PSA) levels in serum and a possible reduction in PC development risk [125]. A randomized follow-up study found that pre-diagnostic aspirin use reduced the risk of lethal PC, and post-diagnostic use improved survival [126].

Meanwhile, in other hormone-dependent cancers, aspirin has not shown relevant results. In BC, there was no apparent effect in decreasing cancer risk or improving survival [127,128]. In ovarian cancer, no association was found between aspirin use and reduced cancer risk, even though a trend toward a protective effect was found with low-dose aspirin [129].

**Table 4:** Aspirin and resveratrol and their application in anti-cancer therapies.

Study Design	Type(s) of Cancer	SOC	CRM	Outcomes	REF.
Multicenter, double-blind, placebo-controlled	None	None	Aspirin and/or NSAIDs	The use of aspirin and NSAIDs reduces serum levels of prostate-specific antigen. Aspirin consumption reduced the risk of prostate cancer development.	[125]
Multicenter, double-blind, randomized, placebo-controlled	None	None	Aspirin 600 mg/d	Obesity-related risk of developing colorectal cancer is reduced after aspirin consumption.	[130]
Randomized, placebo-controlled	None	None	Aspirin 235 mg	Pre-diagnostic use of aspirin lowered the risk of lethal prostate cancer. Post-diagnostic use improved survival compared to placebo.	[126]
Pilot study	None	None	Resveratrol 500 mg	Doses up to 2.5 g of resveratrol were well tolerated in healthy patients and were able to reduce IGF-1 and IGFBP-3 levels.	[131]
Phase 1, non-randomized	CRC	Sx	Resveratrol 0.5 or 1.0 g	Use of resveratrol reduced cell proliferation.	[132]
Phase 1, randomized, double-blind, placebo-controlled	CRC	None	Resveratrol 5 g (SRT501)	The micronized formulation of resveratrol increases apoptosis rate in CRC. No difference in IGF-1, Ki67, AKT, GSK, ERK, JNK, or beta-catenin.	[133]

BC: breast cancer, BTC: biliary tract cancer, CC: Cervix Cancer, CRC: colorectal cancer, CRT: chemoradiotherapy, CT: chemotherapy, DLBCL: Diffuse Large B Cell Lymphoma, EC: endometrial cancer, FMD: fast-mimicking diet, FTC: follicular thyroid cancer, GC: Gastric Cancer, GSH: Glutathione, IGF: insulin-like growth factor, IGFBP-3: IGF Binding Protein 3, MP: Miller–Payne, NSCLC: non-small cell lung cancer, OC: ovarian cancer, OS: overall survival, PaCa: Pancreatic Cancer, PC: prostate cancer, pCR: pathological complete response, PR: partial response, SOC: standard of care therapy, Sx: surgery, TNBC: triple-negative breast cancer.

#### 5.1.4. Resveratrol

Among the natural products related to anti-cancer effects, resveratrol is one of the most studied and the only one considered a CRM. Resveratrol is a stilbene belonging to the polyphenols group, found mostly in grape skin, berries, and in small amounts in red wine and other foods. Its primary activity is as an antioxidant, which can be direct or indirect, scavenging ROS and through the modulation of cellular antioxidant pathways [134,135]. In the anti-cancer effects, resveratrol has shown properties both in vitro and in vivo to inhibit the carcinogenesis stages [136] and as a chemotherapeutic agent [137,138] potentially linked to its pro-apoptotic and anti-proliferative actions.

Even though its promising potential is shown in pre-clinical studies, resveratrol has only been tested in clinical studies for CRC (Table 4). In a phase 1 study of stage 1 CRC, the consumption of 0.5 g or 1 g of resveratrol daily for eight days significantly reduced Ki-67-positive cancer cells [131], illustrating the potential neoadjuvant effect of this CRM. In another phase 1 clinical trial of metastatic CRC, the consumption of 5 g of resveratrol daily increased activated caspase-3 activity by up to 40% without affecting the mTOR and WNT/b-catenin pathways [133].

In healthy volunteers, the consumption of resveratrol reduced IGF1 and IGFBP-3 levels [131], but this effect was not observed in oncologic patients, indicating the need for more trials to characterize the effect of resveratrol on the GH/IGF-1 axis [133].

The principal challenge in using natural products like resveratrol is its poor solubility in water and the low bioavailability after intestinal and liver metabolism, reducing the plasmatic and intra-tumoral levels and, thereby, lowering its efficacy. Luckily, recently, advances have been made in the formulation of delivery systems based on nanoparticles that can enhance the target delivery and bioavailability of resveratrol and its derivates [139].

Once an efficient delivery system for resveratrol is established, the dosage must be determined that provides the best effect with minimal side effects, considering its high intestinal and liver metabolism [140,141] and the potential interactions with other SOC therapies or complementary drugs. More extensive clinical trials are needed to determine the effects of resveratrol in a clinical context. Additionally, more comprehensive pre-clinical and clinical studies on metabolites of resveratrol could address the main challenges of delivery and bioavailability of its parental compound [141,142].

## 6. Discussion

Despite the most recent advances in surgery, chemotherapy, radiotherapy, and biological targeting, cancer remains one of the leading causes of morbidity and mortality. This underscores the necessity for research into complementary therapies that are able to increase disease-free survival, improve quality of life, and potentially lead to complete healing.

Among the most promising complementary therapies, caloric restriction has shown beneficial results in pre-clinical models, including increased lifespan in both non-mammal and mammal models, reduced risk of metabolic diseases, delayed disease progression, and significant anti-carcinogenic and anti-tumoral effects. However, translating these promising preclinical findings to clinical settings has proven challenging.

Firstly, the controlled environment and syngeneic background used in pre-clinical studies reduce the heterogeneity seen in clinical trials. Unlike clinical studies, where patients can be affected by multiple health issues simultaneously, the controlled conditions in preclinical models do not account for the complex interplay of variabilities present in human subjects, potentially rendering CR interventions less effective.

Additionally, the efficacy of CR depends on maintaining a specific caloric intake and fasting duration, which may be challenging for oncologic patients [13,72,74]. The use of SOC therapy, prescribed or non-prescribed drugs, must also be considered when evaluating the potential benefits of a CR intervention. Even though the ketogenic diet may be the most famous and commonly applied CR intervention, its efficacy in an oncologic context remains questionable. This is due to the difficulty in adapting to ketogenic foods, potential early side effects of carbohydrate restriction, and the challenge of achieving and maintaining ketone blood levels that complicate its application [73,74,84]. Hence, all previous factors may interfere with the outcomes, which could explain the lack of conclusive clinical evidence regarding the addition of KD in oncologic patients.

Given these complexities, it is crucial to interpret these results with caution. Most studies reviewed here are phase I/II trials with very limited sample sizes and often involve highly specific cancer types, which may result in heterogeneous responses when applied to other cancer types and comorbidities. Nonetheless, CR interventions might effectively reduce side effects and potentially enhance anti-tumor responses.

To overcome the challenges of implementing a full CR diet in non-compliant patients, whether due to intolerance or difficulty maintaining fasting periods, the use of CRMs might be the most promising intervention alternative. Preclinical models have demonstrated that CRM can replicate most of the molecular and metabolic effects of CR without the need for a restrictive diet. Among CRM, metformin and aspirin are the most well-known, widely used, and have minimal side effects. They are also inexpensive and over-the-counter drugs [90,123,125,143].

After carefully reviewing the current knowledge, the use of CRMs might be recommended when a particular pathway, such as PI3K/AKT/mTOR or IGF1/IGFBP, is upregulated in the tumor and can be modulated by the intervention. However, more evidence is necessary to determine the most appropriate scenarios for the correct CRM.

As a preventive measure, aspirin is the only CRM with sufficient evidence as a chemopreventive agent in patients with an average cancer risk, particularly for CRC. This may be related to its COX-inhibition effects or its role as a CRM. More studies are needed to elucidate its principal mechanism and potential benefits against other cancer types and to determine the optimal timing for its use.

Metformin also shows potential as a chemopreventive agent. Retrospective studies have shown a reduced cancer risk in patients with Type 2 Diabetes Mellitus who use metformin or combine it with other antidiabetic drugs [92,103,143]. Nonetheless, more studies are required to confirm these findings in both diabetic and healthy populations. Furthermore, there is a need to standardize metformin dosage in oncologic studies, as variability in dosing complicates comparisons between trials and expected outcomes.

## 7. Conclusions

In conclusion, caloric restriction interventions and the use of caloric restriction mimetics may serve as beneficial complementary therapies in oncology. Their potential to modulate tumor metabolism, growth, and immune response could enhance the efficacy of standard-of-care treatments. Additionally, these complementary therapies’ protective effects on healthy cells may render them more resistant to cytotoxic therapies, potentially decreasing the side effects of CRT. Thus, integrating these approaches into standard oncologic treatment regimens could provide significant benefits to patients.

## Data Availability

No new data were created or analyzed in this study. Data sharing is not applicable to this article.

## Abbreviations

ABTC: advanced biliary tract cancer, AMPK: AMP-activated protein kinase, BC: breast cancer, CT: chemotherapy, CR: caloric restriction, CRM: caloric restriction mimetic, CRC: colorectal cancer, GH: growth hormone, GR: glucose restriction, HNSS: Head and Neck Squamous Cancer, IF: intermittent gasting, IGF1: insulin-like growth factor 1, LCI: low caloric intake, mTOR: Mammalian Target of Rapamycin, NSCLC: non-small cell lung cancer, NRF2: nuclear factor erythroid 2-related factor 2, NSAID: nonsteroidal anti-inflammatory drug, OS: overall survival, PDCA: pancreatic ductal adenocarcinoma, PR: partial response, PDE3B: phosphodiesterase 3, PI3KS: phosphoinositide 3-kinase, PFS: progression-free survival, PC: prostate cancer, PSA: prostatic-specific antigen, RT: radiotherapy, rapalogs: rapamycin analogs, ROS: reactive oxygen species, STS: short-term starvation, SIRT: sirtuins, TNBC: triple-negative breast cancer, TIL: tumor-infiltrating lymphocyte.
